# Aerosol Pollution from Small Combustors in a Village

**DOI:** 10.1100/2012/956401

**Published:** 2012-05-02

**Authors:** A. Zwozdziak, L. Samek, I. Sowka, L. Furman, M. Skrętowicz

**Affiliations:** ^1^Institute of Environmental Protection Engineering, Wroclaw University of Technology (WUT), Wybrzeze Wyspiańskiego 27, 50-370 Wroclaw, Poland; ^2^Faculty of Physics and Applied Computer Science, AGH University of Science and Technology, 30 Mickiewicza Avenuo, 30-059 Krakow, Poland

## Abstract

Urban air pollution is widely recognized. Recently, there have been a few projects that examined air quality in rural areas (e.g., AUPHEP project in Austria, WOODUSE project in Denmark). Here we present the results within the International Cooperation Project RER/2/005 targeted at studying the effect of local combustion processes to air quality in the village of Brzezina in the countryside north-west of Wroclaw (south western Poland). We identified the potential emission sources and quantified their contributions. The ambient aerosol monitoring (PM_10_ and elemental concentrations) was performed during 4 measurement cycles, in summer 2009, 2010 and in winter 2010, 2011. Some receptor modeling techniques, factor analysis-multiple linear regression analysis (FA-MLRA) and potential source localization function (PSLF), have been used. Different types of fuel burning along with domestic refuse resulted in an increased concentration of PM_10_ particle mass, but also by an increased in various other compounds (As, Pb, Zn). Local combustion sources contributed up to 80% to PM_10_ mass in winter. The effect of other sources was small, from 6 to 20%, dependently on the season. Both PM_10_ and elemental concentrations in the rural settlement were comparable to concentrations at urban sites in summer and were much higher in winter, which can pose asignificant health risk to its inhabitants.

## 1. Introduction

Atmospheric air contains numerous toxic substances as a result of both natural occurrence and anthropogenic activities. Among others, also aerosols are very important. When the air is contaminated by aerosols in general, and heavy metals in particular, it becomes of threat to human health because aerosols can be introduced into the body primarily through the respiratory system and then can enter the human blood circulation rapidly. It leads to an increased risk of certain cardiovascular and pulmonary diseases. Recently, in the USA and Europe, it has been found out that mortality in urban regions and cardiovascular hospital admissions are correlated with the atmospheric levels of fine particles [1–3].

Air particulate matter can also cause chemical deterioration and soiling of structural materials of building, statues, and even paintings. The subject of eventual damages of cultural heritage items through deposition of particles and absorption of gases has already been discussed in the relevant literature [4, 5].

Although the levels of several pollutants have evidently decreased in many urban areas in Europe, this trend seems to be questionable in rural and semirural areas. It is observed that people in urban areas are moving to favor more healthy rural living, thus it is important to obtain some information on the levels and chemical composition of airborne particulate matter containing trace metals in rural settlements. The Technical Cooperation Project RER/2/005 on “Characterizing Seasonal Variations in Elemental Particulate Matter Concentrations in European Urban and Rural Areas under Different Climatic Conditions” was started in 2009 as a dual site approach with one urban and one rural site. The urban site should be representative for “urban background”, that is, residential area without direct influence of traffic. The acceptable rural location should be situated in a small town/village. The settlement should be characterized by use of solid fuel for domestic heating. The purpose of the campaigns was to assess the effect of local combustion processes on air quality. Over two-year period (2009–2011), continuous monitoring of PM_10_ with consequent elemental characterization of the aerosols was conducted for two weeks per site per season for both years. Additionally, receptor modeling techniques have been used to identify and quantify the potential sources contributing to PM_10_ mass.

## 2. Materials and Methods

**Figure 1:**
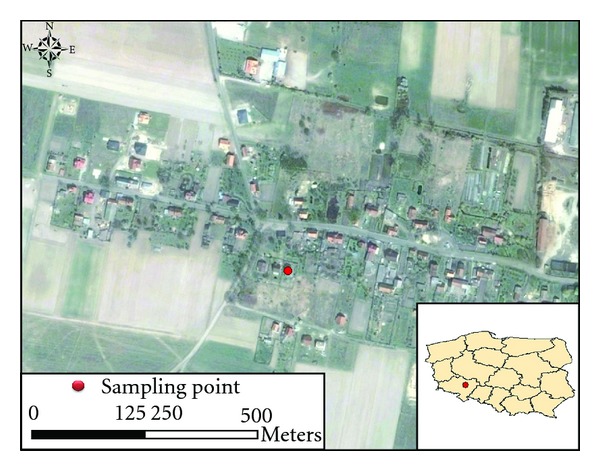
Location of the sampling site in Brzezina, Poland (source: “Bing Maps Aerial").

Brzezina (51°12′12′′N,16°49′38′′E) is a small village of approximately 350 inhabitants, surrounded by agricultural areas, and with no local industry. The nearest industrial site is Wroclaw (634 000 inhabitants), approximately 20 km southeast and copper mining and processing plants, about 50 km to the west and northwest of the site. Brzezina was selected as a suitable site because it is a typical bedroom community with all houses equipped with small combustors for burning different kind of fuels. Measurements were conducted in a garden at the outskirts of the village (Figure 1). The nearest surroundings of the measurement site were meadows and private detached houses with gardens. The small streets in a village were mainly used by cars for a few vehicles per hour during the day.

Aerosol PM_10_ was collected for two weeks in August 2009, July/August 2010 and in February 2010, February 2011 on a daily basis with a TCR TECORA Sampler (Model Charlie HV, Italy). The sampling rate was set at 2.3 m^3^/h. Sampling substrates were Teflon—membrane 47 mm diameter filters (Whatman PTFE, 2 um pore size). Filters were weighted to evaluate the PM_10_ mass on a microbalance in a controlled clean room. Filter weight before and after sampling was obtained as the average of three measurements. Concentrations of following elements were measured: K, Ca, Cr, Mn, Fe, Cu, Zn, Br, Pb, and As. Chemical analyses were performed by X-Ray Fluorescence Analysis in the laboratory of Faculty of Physics and Applied Computer Science, AGH university of Science and Technology, Krakow, Poland. The University is equipped with the Energy Dispersive X-Ray Fluorescence Spectrometer. The details of sampling and the accuracy of analytical methods are given in [6]. In our source apportionment studies, only elements that were above the detection limit (DL) in over 50% of the samples were included.

For further identification of PM_10_ emission sources, PCA analysis (STATISTICA software) was applied to the element concentration data obtained in the rural site. Then, multilinear regression analysis was performed for evaluation of the contribution of each source group to the PM mass concentration. The method was developed by Thurston and Spengler [7] in which FA was conducted, absolute zero values calculated and applied to give absolute FA scores, followed by a regression of the mass to apportion PM_10_ to source categories and locations under study. Details of source apportionment methodology and results are presented in Almeida et al. [8, 9].

Meteorological parameters measured during the period of the field studies were wind speed and direction, temperature, relative humidity, pressure and precipitation, which were obtained from Wroclaw Airport, Starachowice about 10 km in the south direction. The backwards trajectories of the air masses were calculated by the HYSPLIT model [10] run by NOAA (http://www.arl.noaa.gov/).

## 3. Results and Discussion

### 3.1. General Weather Conditions during Field Studies

Main meteorological conditions for the following field studies are shown in Table 1. Summer 2009 as well as summer 2010 was characterised by sunny weather and high air temperature. In winter 2010 and 2011, the weather conditions were mostly sunny, however, with foggy and snowy episodes. During summer seasons, the maximum and minimum temperatures were found to be similar; the differences did not exceed 3°C. The relative humidity and wind conditions were also comparable for two measurement periods. During the winter periods, the temperature was noticeably lower in 2010 than 2011 and with higher frequency of precipitation events (snow). The winter measurement period in 2011 was dominated by stronger western winds, thus with greater air ventilation potential than in winter 2010. Low variable winds prevailed for most of measurement days in 2010.

**Figure 2:**
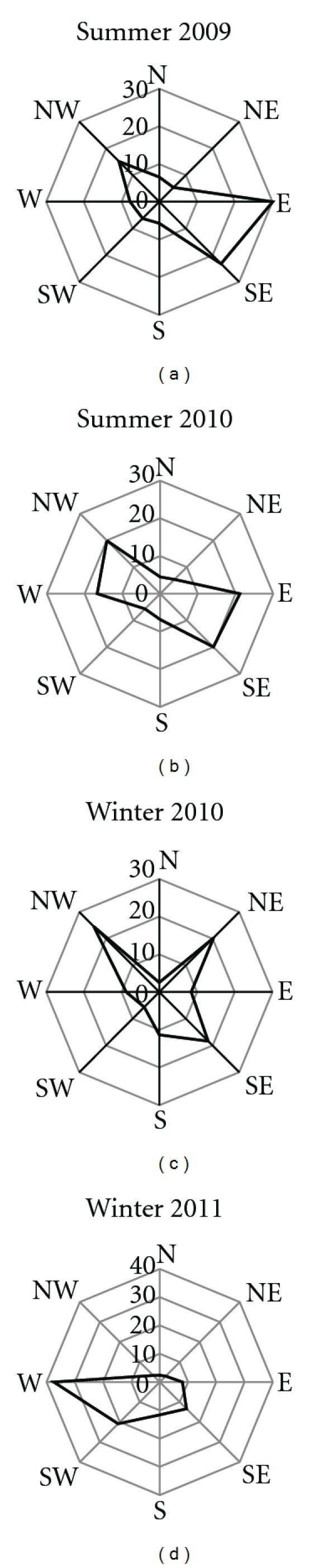
Wind direction frequency (%) for summer 2009 (a), 2010 (b) and winter 2010 (c), 2011 (d) field studies.

Figure 2 shows the frequencies of wind directions—calculated on the basis 1-hour wind data for summer and winter periods. Wind roses are a popular way to represent local transport information. The data show that (1) eastern and southeastern winds dominated in summer 2009; (2) comparable southeastern and northwestern components occurred during summer 2010; (3) winds distinctly changed direction during winter 2010; (4) western winds dominated in winter 2011. Different wind conditions give one of the explanations for the strong variation of the PM_10_ concentration.

### 3.2. PM_**10**_ and Elemental Concentrations

Average daily PM_10_ and elemental concentrations are summarized in Table 2. As shown in Table 2, very high concentrations of PM_10_ were observed during the cold seasons, 91.2 and 54.7 *μ*g/m^3^in 2010 and 2011, respectively. The PM_10_ concentrations for 2009 and 2010 summer field studies were considerably lower than observed during the cold seasons, (all summer data: 21.6 ± 8.0 *μ*g/m^3^; all winter data: 75.7 ± 43.3 *μ*g/m^3^). The strong seasonal variability was also monitored in rural sites in the Czech Republic [11, 12] and was explained by the local emissions from heating facility sources during the winter months. On the contrary in Austria, at the rural site 30 km west of Vienna, the concentrations of PM_10_ showed no strong variability during the seasons of the year and the long-term average was 21.1 *μ*g/m^3^ [13]. However, Gomiscek et al. [14] reported that concentrations of PM_10_ were above 60 *μ*g/m^3^ in some episodic situations at the same rural site in Austria.

By comparing the present results with data collected in urban background air monitoring stations in Wroclaw [15], we could observe comparable PM_10_ concentrations in nonheating seasons, while values measured in Wroclaw during heating seasons were much lower than those observed in Brzezina and ranged between 28 and 32 *μ*g/m^3^. High PM_10_ concentrations in Brzezina, higher than in the city, were clearly governed by the local emission from heating facility sources and to some extent to traffic. Particularly, that levels remained high during the whole winter sampling periods. The highest PM_10_ mass concentrations were monitored in February, 2010. Temperature remained very cold on these days (minimum: −14°C), thus PM_10_ peak concentrations were most likely due to increased emission from heating sources. Even observed isolated snow events in winter 2010 with low wind speed (wind gust) did not reduce high dust concentrations. This phenomenon was also indicative for the local origin of PM_10_. Generally storms reduce air pollution; however, the discussion of this problem is out of the scope of this paper. 

 The European Union air quality daily PM_10_ standard is 50 *μ*g/m^3^, that can be exceeded a maximum of 35 days per year. 11 daily PM_10_ concentrations were higher than the limit value, which represents almost 80% of the sampling days in February 2010. The corresponding data for February 2011 was 45% (6 cases). In the case of trace elements, only some are discussed at policy level in the European Union. Currently, there are annual target values for Ni, As, Cd (they enter into force 1.1.2012) and a limit value for Pb. These elements should not exceed the annual standard of 20 ng/m^3^, 6 ng/m^3^, 5 ng/m^3^, and 0.5 *μ*g/m^3^, respectively. For Pb, the standard was much higher than the observed concentrations. Cd and Ni were not detected in the samples. Nevertheless, the reported levels for As in some samples exceeded 6 ng/m^3^.

 As can be seen from Table 2, there were distinct differences in trace element concentrations associated with PM_10_ among four measurement periods. Concentrations rose in winter seasons, reaching values up to 4 times higher than in summer. For example, the average concentrations of Pb were 12,5 ng/m^3^ and 29.8 ng/m^3^ in summer studies, and 85.9 and 52.0 ng/m^3^ in winter periods. In the case of Zn, the mean concentrations were 44 and 26 during summertime, and 199 and 119 ng/m^3^ in winter studies. Strong fluctuations of the concentrations can be demonstrated by the standard deviation divided by the mean. As and Cr were the elements with a coefficient above 1.0. The average concentrations of these elements should be considered as indicative since there was a high uncertainty on the XRF values (they were often below the detection limit). Nevertheless, they appeared episodically in high concentrations in ambient aerosol, indicating that specific sources were at play. Different wind conditions together with variable other weather parameters (Table 1) could only partly explain significant variations in observed elemental concentrations.

### 3.3. Receptor Modeling Techniques

Seasonal trends in PM_10_ and some trace elements concentrations can suggest that local combustion sources are predominantly active during wintertime. Some simple techniques, as factor analysis (FA), allow deriving information whether a combustion is the only pollution source in rural areas and to which extent trace metals are originating from this source.  Table 3 gives the matrixes of the loadings after rotation for the data from the following field campaigns. The matrixes can be used to combine sources with the rotated factors. There are strongly correlated compounds observed for each factor. Two sources accounted for 77% of the total variance for the summertime and 76% and 87% for the wintertime data in 2010 and 2011, respectively. The first factor (F1) explained that most of the variance (51 ÷ 64%) had high loadings of most elements investigated, however, with their different combination. We have named it regional background dust as the elements: Ca, K, Mn, Fe, Zn, Cu, Pb, Br, with the highest factor loadings can be related to a dust mixture of different origin and represent the main types of the pollution sources in the region. The elements Ca, K, and Fe are believed to contribute to the soil component in summer and fly ash in winter [16, 17]. Resuspension of roadway dust is also dominated by crustal elements Si, Fe, Ca, Na, Mg, Al, and K [18]. Metals related to traffic emission are Cu, Zn, Pb, and Br [18–20]. We can notice an increase in the concentrations of these elements in winter periods, which correspond to a decrease in the temperature measured. Much higher concentrations of K, Zn, Cu, Pb, Br, the elements characteristic for different kind of combustion processes, demonstrate that local sources, including biomass/refuse burning, coal, and fuel combustion are very important in the winter. Generally, it is very difficult to distinguish between soil and fly ash contribution. However, resuspension of soil particles during wintertime, particularly when the ground is frozen, seems to be less important process than combustion. The second factor (F2) explained from 13 to 30% of the variance and was probably associated with industrial pollution sources because Cu, Pb, Cr, Mn, Fe, and As are known to have industrial origin. What is interesting, these metals can represent different types of pollution sources. 

**Figure 3:**
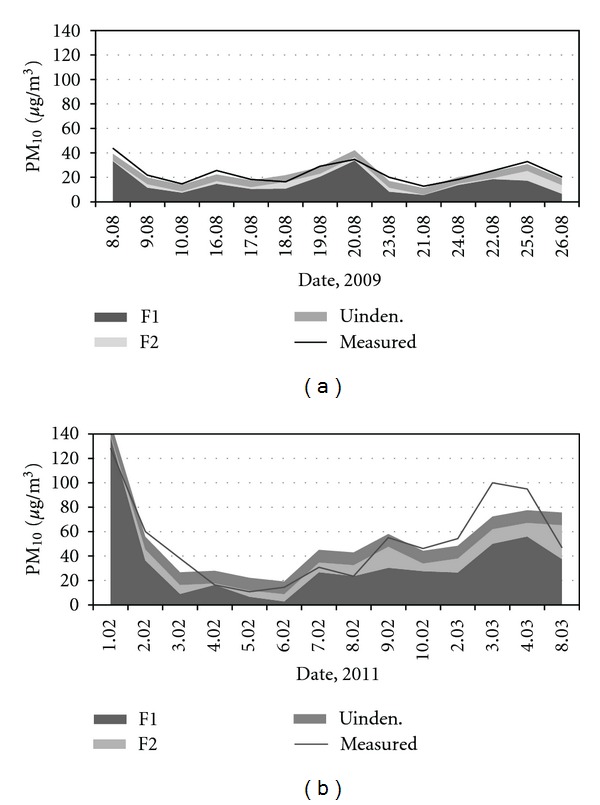
Daily source contributions to PM_10_ obtained by FA-MLRA and measured by gravimetry for summer 2009 and winter 2011.

Daily contributions of each source to ambient PM_10_ in Brzezina were estimated using FA-MLRA methodology [7]. Figure 3 shows an example of time series of the contributions of the identified sources for the summer period in 2009 and winter in 2011. On the basis of daily contributions, the average mass contributions of each source (SC—source contribution) were calculated and the results are summarized in Table 3 (the last raw). It can be seen in Table 3 that the average mass contribution of distant industrial emission sources was 12% and 15% in the summer 2009, 2010 and 6% and 20% in the winter 2010, 2011. Background dust contributed with 61% or 31% of total PM_10_ mass during the summer campaigns. However, nonidentified sources contributed with about 55% in the summer 2010. It was probably due to high concentrations of sulphates, nitrates, and a certain amount of organic carbon which are characteristics secondary pollutants and are formed, especially in the summer, when solar radiation and the temperature are high. Secondary pollutants are usually indicative for a long distance transport. However, these pollutants were not measured.

Local combustion sources contributed with 79% in winter 2010 and 55% in winter 2011, reflecting serious local problem associated with PM_10_ air pollution. Generally good compatibility was found between the measured PM_10_ concentrations by gravimetry and estimated by FA_MLRA (Figure 3). The squared correlation coefficient, *R^2^*, was 0.80 and 0.72 for summer and winter data, respectively.

**Figure 4:**
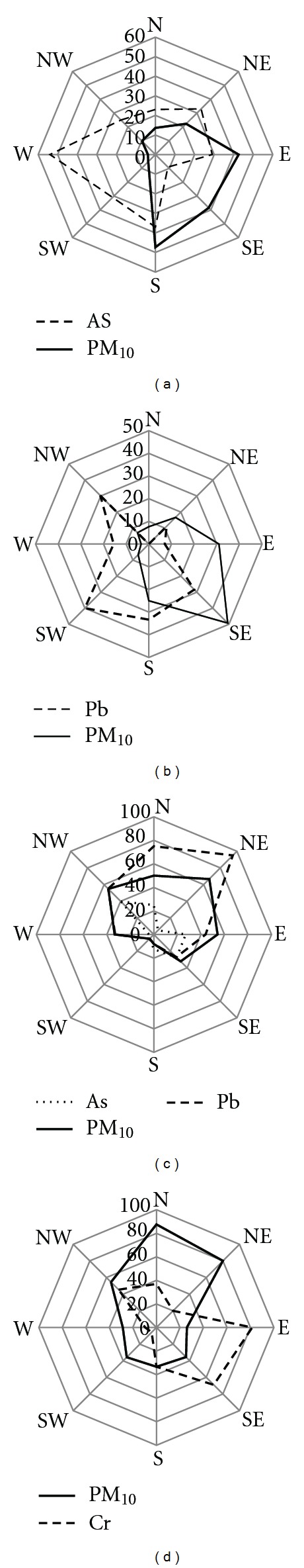
PSL plots (%) of: (a) PM_10_ and arsenic during summer 2009; (b) PM_10_ and Pb during summer 2010; (c) PM_10_ and As, Pb during winter 2010; (d) PM_10_ and Cr during winter 2011.

**Figure 5:**
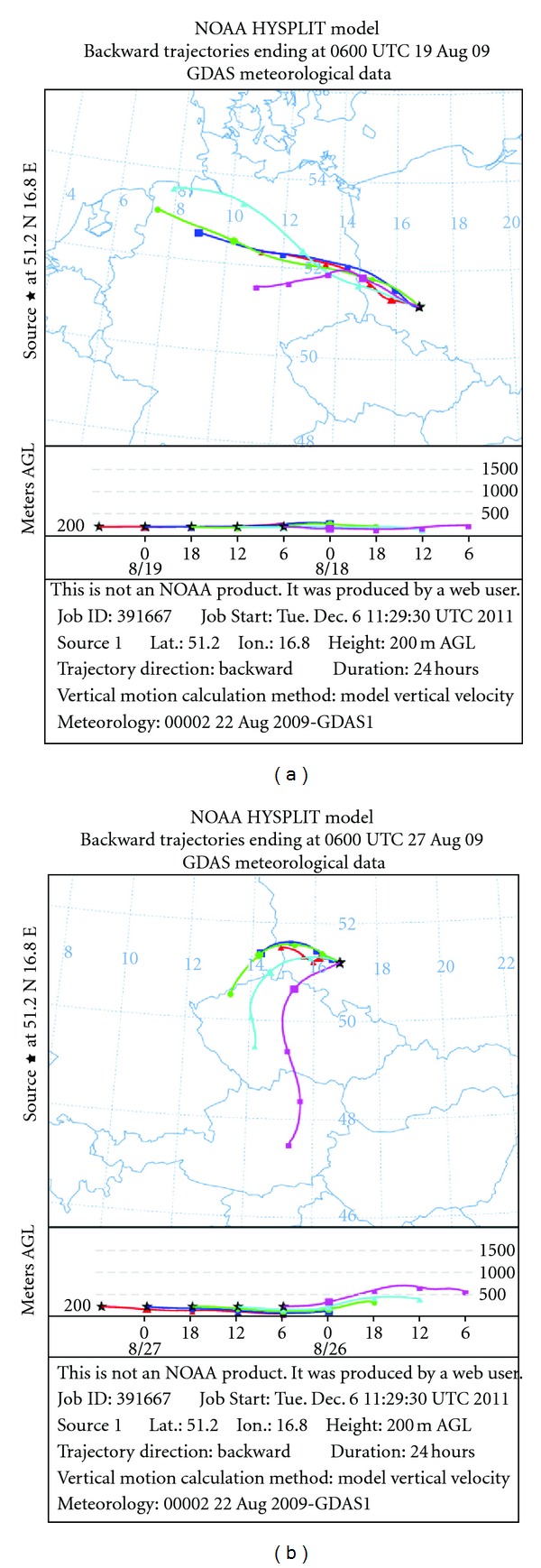
24 h backward trajectories for the days with the highest As concentrations (19 August 2009 and 27 August 2009) arriving at the rural site at 06:00 UTC (08:00 LT).

 Now, the question is what industrial sources could contribute to total PM_10_ mass. Trajectory cluster analysis, conditional probability functions (CPF), and potential source contribution functions (PSCF) have been successfully used to identify transport paths and source areas [21–23]. These techniques demand long-term data, but we had only two weeks pollutant concentration time series. Thus, we defined the potential source localization function (PSLF) similarly to CPF [21]:

(1) $${\text{PSLF}}_{ij}=\frac{n_{ij}}{m_{j}},$$

where, *n*_*ij*_ is the number of cases with wind, “*j*” direction connected with high daily concentrations of the species “*i*” (the 75th percentile); *m*_*j*_—is the total number of cases with “*j*” wind direction. Thus, the PSL function can be interpreted as a probability describing the local wind direction as aprobable source localization direction. At first, we calculated the wind direction statistics based on one-hour data for each day during field studies. Wind directions came from Wroclaw Airport, Starachowice, about 10 km in the south direction. Then, we extracted days with high measured values of PM_10_ and selected elemental concentrations (above the 75th percentile). At the end, we computed the backward trajectories using the HYSPLIT-4 model [10] for the episodic days and assessed the pollution source areas most likely to be upwind of the receptor.  Figure 4(a) presents the PSL plots of PM_10_ and arsenic during summer 2009. PM_10_ concentrations greater than 28 *μ*g/m^3^ (the 75th percentile) were observed more frequently for the south-eastern wind sector and corresponded to wind speed values less than 2 m/s. Although southeastern and eastern wind dominated (see the wind rose in Figure 2), the PSL plots for PM_10_ show that the whole south-east sector was associated with elevated PM_10_ levels. Arsenic in higher concentrations (above 4,8 *μ*g/m^3^—the 75th percentile) was more frequently connected with south-western winds which confirms that its origin is different than the main PM_10_ components. Arsenic can be released to air from various industrial sources (e.g., coal combustion, smelter, and mining activities) and pesticide application. Arsenic episodes on August, 18/19 and 26/27, corresponded to trajectories presented in Figure 5. 36-hour back trajectories were calculated by the HYSPLITT model for air masses ending over Brzezina at 200 m height level. From the data presented in Figure 5, it becomes obvious that that the recorded high values of As concentrations are probably due to dust transported from western directions. The nearest stationary source is a copper smelter located upwind (W) 50 km of the receptor (Legnica copper smelter). 

**Figure 6:**
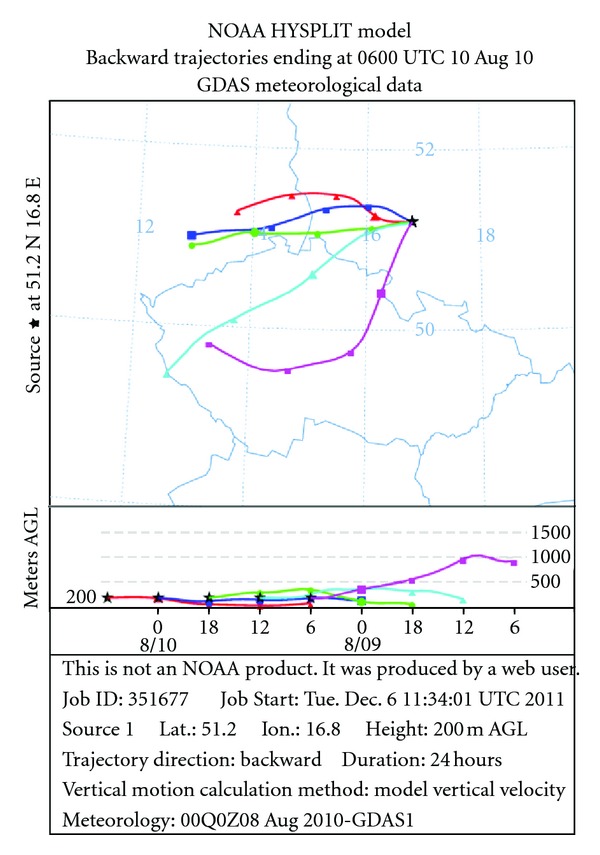
24 h backward trajectories for the day with the highest Pb concentrations (10 August 2010) arriving at the rural site at 06:00 UTC (08:00 LT).

 The highest PM_10_ concentrations measured during the summer period in 2010 (Figure 4(b)) were more frequently connected with the south-eastern wind sector with the speed values below 2 m/s. Arsenic was not detected, but lead, which represents the other origin (together with Cu) than the remaining measured PM_10_ components, was mainly advected with air masses from the south and south-western direction. There are no appropriate emissions inventories and thus, it is difficult to appoint the likely significant Pb and Cu sources which can include nonferrous metal smelting and processing or solid waste incineration. Lead episodes on August 10, 2010 corresponded to trajectories presented in Figure 6.

 In winter 2010 the situation was different.  Figure 4(c) shows that air masses contributing to worst-case arsenic concentrations tend to originate to the southeast and northwest of the village. However, it seems that very high As concentrations (above 10 *μ*g/m^3^) can originate rather from local sources than regional ones, and probably from man-made local combustion devices. This source category includes incineration of various types of domestic refuse which is so common in small villages. Very low temperatures and low wind speeds resulted in small vertical mixing and in very high As concentrations as well as other elements. The diurnal variations of other PM_10_ components were similar to those of PM_10_. As an example, Pb is shown in Figure 4(c). PM_10_ episodes, as well as Pb, were more frequently monitored when wind was blowing from the north and north-east. The PSL plots for Br and Zn were very similar to that of Pb and are not shown here. It identifies the whole village Brzezina as the potential PM_10_ and Pb, Br, Zn pollution source and supports the idea that combustion processes are the major sources of PM_10_ and some elements.

Most interesting results were obtained for winter 2011 data. Although the western winds prevailed during this period (Figure 2(d)), major contributing directions for PM_10_ were from north as shown in Figure 4(d) similarly to 2010 data (Figure 4(c)). The most important wind directions for the elevated concentrations of Cr (above 20 *μ*g/m^3^—the 75th percentile), which represents the industrial source (together with Mn and Fe), were from the east and southeast. This direction appointed Wrocław with some metallurgical industrial plants. However, pollution source recognition is a very subjective process and strongly influenced both by the interpreter of the results and knowledge of the site.

## 4. Conclusions

Generally, the concentrations of PM_10_ at the rural site were high and showed strong variability during the seasons of the year exceeding the European Union air quality daily PM_10_ standard of 50 *μ*g/m^3^ in winter periods. The seasonal variation in the rural atmosphere was clearly inducted by the local emissions from winter which was confirmed by the potential source localization calculations. The PSL plots identified the whole village as the potential contributor of PM_10_ mass and Pb, Br, Zn, As concentrations and supported the idea that combustion processes are the major sources of PM_10_ and some elements in wintertime.

The evidence on regional transport of some toxic metals was provided for each season. However, it was found difficult to appoint the potential emission sources using only wind rose data. Two receptor modeling techniques PSLF and FA-MLRA were applied, and the results obtained were in general in good agreement, showing that some metals in higher concentrations were more frequently connected with different wind direction than the main PM_10_ components that confirmed their different origin as appointed by FA.

Air pollution problem is not only typical for the village under interest. Wood burning along with domestic refuse and the poorest and cheapest types of fuel is probably widely present in individual heating houses not only in Poland. This phenomenon undoubtedly contributes to high concentrations of PM_10_ and the high abundance of many elements in air in the winter which can pose serious health problems.

## Figures and Tables

**Table 1:** Meteorological conditions during the field studies.

Parameter	Date			
	8–10.08; 16–27.08.09	29.07–12.08.10	10–24.02.10	1 ÷ 11.02; 2 ÷ 5.03.11
*t*_max⁡_, °C	26 ÷ 33	26 ÷ 34	−2 ÷ 10	−4 ÷ 12
*t*_min⁡_, °C	9 ÷ 16	12 ÷ 18	−14 ÷ 0	−10 ÷ 10
Humidity, %	67 ÷ 82	62 ÷ 94	74 ÷ 92	60 ÷ 96
Precipitation events	17, 21/22	3, 7/8	11, 13, 15, 20, 21, 23, 24	4.02
Wind conditions	E-SE; <2 m/s	E-SE, <2 m/s	Variable, <2 m/s	W, <5 m/s

**Table 2:** Average daily PM_10_ and elemental concentrations with standard deviation at the rural site (Brzezina) for following measurement periods.

Element	Summer 2009	Summer 2010	Winter 2010	Winter 2011
K, ng/m^3^	324 ± 170	401 ± 98	648 ± 327	442 ± 346
Ca, ng/m^3^	474 ± 342	370 ± 202	376 ± 145	505 ± 342
Cr, ng/m^3^	< DL*	< DL*	30 ± 32	22.3 ± 18.9
Mn, ng/m^3^	37.1 ± 21.2	15.6 ± 11.6	44.2 ± 37.3	34.8 ± 21.8
Fe, ng/m^3^	630 ± 367	306 ± 198	500 ± 368	558 ± 348
Cu, ng/m^3^	10.9 ± 5.1	10.7 ± 3.2	32.2 ± 19.3	16.1 ± 9.5
Zn, ng/m^3^	44 ± 33	26 ± 14	199 ± 118	119 ± 93
Br, ng/m^3^	7.5 ± 1.9	2.8 ± 1.6	28.3 ± 16.3	19.4 ± 12.1
Pb, ng/m^3^	12.5 ± 8.9	29.8 ± 20.7	85.9 ± 52.8	52.0 ± 45.5
As, ng/m^3^	3.5 ± 4.4	< DL*	4.3 ± 5.17	4,9 ± 5.4
PM_10_, *μ*g/m^3^	23.9 ± 8.4	19.4 ± 7.0	91.2 ± 49.5	54.7 ± 38.8

*Below detection limit.

**Table 3:** VARIMEX normalized rotated factor loadings for a factor analysis on Brzezina PM_10_ data set. Loadings for which the absolute value is greater than 0.700 are indicated in italic.

	Summer 2009			Summer 2010			Winter 2010			Winter 2011		
El.	F1	F2	Com.	F1	F2	Com.	F1	F2	Com.	F1	F2	Com.
K	*0,92*	0,10	*0,85*	*0.84*	0.29	0.90	*0,79*	0,42	0,80	*0,95*	0,11	0,97
Ca	*0,98*	0,08	*0,97*	*0.86*	0.33	0.96	0,63	0,54	0,68	*0,78*	0,23	0,94
Cr	0,47	0,05	0,23	—	—	—	*0,71*	0,05	0,51	0.10	*0,95*	0,89
Mn	*0,95*	0,02	0,91	*0.88*	0.05	0.80	*0,87*	0,22	0,81	0,39	*0,88*	0,98
Fe	*0,96*	0,21	0,96	*0.88*	0.33	0.94	*0,90*	0,21	0,86	0,65	*0,73*	0,98
Cu	0,48	0,54	0,53	0.37	*0.91*	0.95	*0,82*	0,07	0,68	*0,70*	0,54	0,96
Zn	*0,92*	0,03	0,84	*0.78*	0.29	0.94	*0,95*	0,16	0,94	*0,95*	0,22	0,98
Br	*0,86*	0,20	0,77	0.36	0.38	0.43	*0,93*	0,11	0,88	*0,88*	0,32	0,94
Pb	*0,87*	0,12	0,80	0.16	*0.94*	0.96	*0,73*	0,45	0,74	*0,93*	0,15	0,98
As	0,09	*0,95*	0,91	—	—	—	0,07	*0,85*	0,74	*0,82*	0,02	0,90

Var.,%	64	13	77	57	20	77	61	15	76	57	30	87

S.C.,%	61	12		31	15		79	6		55	20	

Com. is the communality: the proportion of a variable's variance explained by a factor structure.

Var., % is the total variance explained by a factor in percent.

S.C., % is average source contribution to PM_10_ mass concentration in percent.
